# Heterostructured ZnFe_2_O_4_/Fe_2_TiO_5_/TiO_2_ Composite Nanotube Arrays with an Improved Photocatalysis Degradation Efficiency Under Simulated Sunlight Irradiation

**DOI:** 10.1007/s40820-017-0169-x

**Published:** 2017-11-16

**Authors:** Kun Xiong, Kunzhou Wang, Lin Chen, Xinqing Wang, Qingbo Fan, Jérémie Courtois, Yuliang Liu, Xianguo Tuo, Minhao Yan

**Affiliations:** 1 State Key Laboratory Cultivation Base for Nonmetal Composites and Functional Materials, Southwest University of Science and Technology, Mianyang, 621010 People’s Republic of China; 2 Sichuan University of Science and Engineering, Zigong, 643000 People’s Republic of China

**Keywords:** Titanium dioxide nanotube arrays, Zinc ferrites nanocrystals, Pseudobrookite, Photocatalysis, Methylene blue, Heterojunction

## Abstract

To improve the visible light absorption and photocatalytic activity of titanium dioxide nanotube arrays (TONTAs), ZnFe_2_O_4_ (ZFO) nanocrystals were perfused into pristine TONTA pipelines using a novel bias voltage-assisted perfusion method. ZFO nanocrystals were well anchored on the inner walls of the pristine TONTAs when the ZFO suspensions (0.025 mg mL^−1^) were kept under a 60 V bias voltage for 1 h. After annealing at 750 °C for 2 h, the heterostructured ZFO/Fe_2_TiO_5_ (FTO)/TiO_2_ composite nanotube arrays were successfully obtained. Furthermore, Fe^3+^ was reduced to Fe^2+^ when solid solution reactions occurred at the interface of ZFO and the pristine TONTAs. Introducing ZFO significantly enhanced the visible light absorption of the ZFO/FTO/TONTAs relative to that of the annealed TONTAs. The coexistence of type I and staggered type II band alignment in the ZFO/FTO/TONTAs facilitated the separation of photogenerated electrons and holes, thereby improving the efficiency of the ZFO/FTO/TONTAs for photocatalytic degradation of methylene blue when irradiated with simulated sunlight.

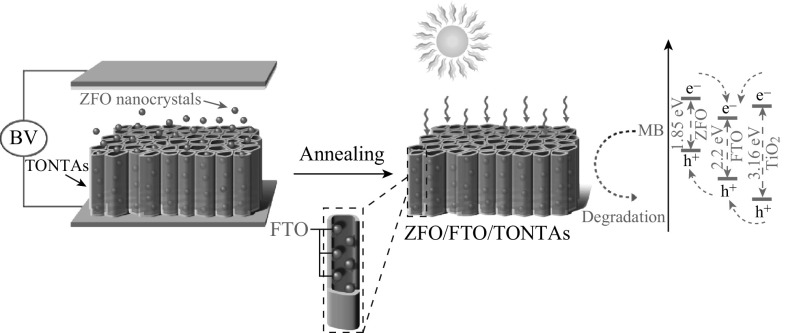

## Highlights

- ZnFe_2_O_4_ nanocrystals were perfused into pristine TiO_2_ nanotube array pipelines using a novel bias voltage-assisted perfusion method.
- Novel heterostructured ZnFe_2_O_4_/Fe_2_TiO_5_/TiO_2_ composite nanotube arrays were obtained with staggered type II band alignment at the ZnFe_2_O_4_/Fe_2_TiO_5_ interface and type I band alignment at the Fe_2_TiO_5_/TiO_2_ interface.
- Visible light absorption and the photocatalytic degradation efficiency of methylene blue were significantly improved upon irradiation with simulated sunlight.

## Introduction

Semiconductor photocatalysts have attracted considerable research interests because of their potential value in environmental remediation [1–5]. As an excellent UV light-driven photocatalyst, nano-TiO_2_ powders are already commercially available (Evonik Degussa P25). Recently, TiO_2_ nanotube arrays (TONTAs) were found to possess superior electron percolation pathways for charge transfer than randomly arranged TiO_2_ nanocrystals, which is probably due to their unique structural arrangement [6, 7]. Therefore, TONTAs have been widely investigated for hydrogen production via water splitting [8–10], photocatalytic degradation of organic pollutants [11–13], dye-sensitized solar cells [14–17], and photoelectrodes [18–20]. However, because of the wide bandgap of TiO_2_ (3.2 eV for anatase; 3.0 eV for rutile), it absorbs light in the UV region, which only occupies 4–5% of the total solar spectrum. This results in lower solar energy utilization [21]. Therefore, many methods have been investigated to improve the visible light absorption of TONTAs.

Visible light absorption by TONTAs can be enhanced by metal (Ag [22], Au [23, 24], and Pd [25]) and nonmetal (N [26, 27], C [28, 29], F [30–32], and B [33, 34]) doping, but this approach remains challenging because of carrier recombination centers. Based on heterojunction energy band theory, the recombination of photogenerated electrons and holes can be effectively reduced when a staggered type II band alignment is formed at the interface of TONTAs and narrow bandgap semiconductors (such as CdS [35–37], CdSe [38, 39], RuO_2_ [40, 41], and NiO [42, 43]).

In recent years, ZnFe_2_O_4_ (ZFO) has attracted considerable attention because of its narrow bandgap (1.86 eV), which can help it to harvest approximately 46% of sunlight [44, 45]. In contrast to pure TiO_2_ nanoparticles, ZFO/TiO_2_ composite nanoparticles exhibited a better activity in the photodegradation of methyl orange, but their energy conversion efficiency remained relatively low [46]. Furthermore, the visible light-induced photocatalytic activity could be further enhanced when ZFO nanocrystals were anchored on the inner walls of the TONTAs [21]. Additionally, the ZFO/TONTA composite was investigated and required no sacrificial agent to consume the photogenerated electrons. Charge carriers that were excited from TiO_2_ and were transferred to ZFO could recombine, which lowered the photocatalytic efficiency for the degradation of methyl orange [47]. Similar effects were found when BiFeO_3_ nanocrystals were anchored on the inner walls of TONTAs. Sarkar et al. [48] found that these heterostructures facilitated separation of photogenerated electrons and holes to form more hydroxyl radicals ($^{{ \cdot }} {\text{OH}}$). Zhu et al. [49] reported that lattice-matched pseudobrookite (Fe_2_TiO_5_, FTO) could be grown on the surface of TiO_2_ via solid-state reactions between Fe_2_O_3_ and TiO_2_. In contrast to TiO_2_, FTO has a relatively narrow bandgap (2.2 eV) [50]. Liu et al. [51] prepared an ultrathin FTO layer on the inner walls of TONTAs and showed that visible light absorption of the FTO/TiO_2_ composite nanotube arrays and the associated energy conversion efficiency were significantly improved.

Herein, we describe a novel heterostructured ZFO/FTO/TiO_2_ composite nanotube array (ZFO/FTO/TONTAs). No studies have yet reported the preparation of ZFO/FTO/TONTAs or their photocatalytic performances. In this work, ZFO nanocrystals were first perfused into pristine TONTA pipelines using a bias voltage. They were then annealed to form ZFO/FTO/TONTAs. The phase composition, microstructure, and photocatalytic performance of ZFO/FTO/TONTAs were investigated, and the photocatalytic enhancement mechanism was also discussed.

## Experimental Section

### Synthesis of TONTAs and ZFO Nanocrystals

TONTAs were prepared using the electrochemical anodization method. Prior to anodization, the commercially purchased Ti foils (40 × 50 × 1 mm^3^, purity > 99.5%) were physically and chemically polished. The Ti foils were anodized in a two-electrode cell, and Pt-plated Ti (Pt/Ti) foil was used as the cathode. The electrolyte was composed of 1.5 wt% ammonium fluoride, 91 vol% ethylene glycol, and 9 vol% deionized (DI) water. When the anodization voltage was held at 60 V for 2 h, the largest TONTAs (diameter ≈ 180 nm) were obtained.

The hydrothermal method was used to prepare ZFO nanocrystals, as described in our previous study [52]. Briefly, Zn(NO_3_)_2_·6H_2_O (Sigma-Aldrich) and Fe(NO_3_)_3_·9H_2_O (Sigma-Aldrich) were completely dissolved in 60 mL of DI water, corresponding to final concentrations of 20 and 40 mM, respectively. The pH value was adjusted to 10 by dropwise addition of an ammonia solution (25 wt%) to obtain a well-dispersed brown dispersion. The samples were transferred into a 100-mL Teflon autoclave, sealed, and heated at 170 °C for 6 h. Then, the brown precipitates were collected and transferred to a new beaker. The obtained products were carefully rinsed three times with DI water.

To minimize soft aggregation of ZFO nanocrystals, concentrated nitric acid (60 wt%) and DI water were added at a volume ratio of 1:3 and stirred with a magnetic stirrer. This process was repeated three times. The stirring times were 30 (first time), 20 (second time), and 10 min (third time). A magnet was used to deposit the as-prepared ZFO nanocrystals on the bottom of the beaker, and the supernatant liquid was removed. With magnetic stirring, the ZFO nanocrystals were washed with acetone for 30 min to remove the residual nitrate ions and were re-dispersed in DI water to obtain a bright brown liquid with a ZFO concentration of 0.75 mg mL^−1^.

### Preparation of ZnFe_2_O_4_/Fe_2_TiO_5_/TiO_2_ Composite Nanotube Arrays

**Fig. 1:**
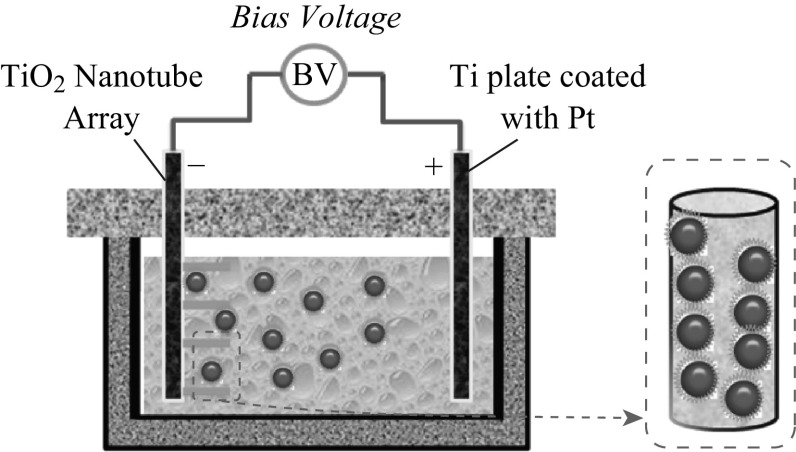
Schematic representation of the bias voltage-assisted perfusion process

A novel bias voltage-assisted perfusion method was used to anchor the as-prepared ZFO nanocrystals on the inner walls of the pristine TONTAs (as shown in Fig. 1). The TONTA/Ti foil and Pt/Ti foil were separately used as the cathode and anode, respectively. The ZFO nanocrystals were well perfused into pristine TONTA pipelines under a 60-V direct current bias voltage. The perfusion time and concentration of ZFO nanocrystals were 1 h and 0.025 mg mL^−1^, respectively. When the perfusion process was finished, the TONTA/Ti foil was repeatedly rinsed with DI water and air-dried. They were subsequently transferred into a furnace and annealed under varying temperatures (650, 750, and 850 °C) for 2 h in air. Finally, a blade was used to remove the as-prepared ZFO/FTO/TONTAs from the Ti foil.

### Characterization of the Samples

The phase composition of the samples was characterized by X-ray diffraction (XRD, X’Pert PRO, PANalytical, the Netherlands) using a Cu *K*α source (*λ* = 1.5406 Å). The morphology of the samples was observed using field emission scanning electron microscopy (FE-SEM, ZEISS Ultra 55, Germany) and high-resolution transmission electron microscopy (HR-TEM, LIBRA 200FE, ZEISS, Germany). The chemical compositions of the samples were determined by X-ray photoelectron spectroscopy (XPS, XSAM 800, Kratos, UK), and the binding energies were normalized to the signal for adventitious C1*s* at 284.8 eV.

The hydrodynamic diameter (*D*_H_) of the as-prepared ZFO nanocrystals in dispersion was monitored by a dynamic light scattering (DLS) technique using a multi-angle particle size and zeta potential analyzer (Brookhaven NanoBrook Omni, USA). The specific surface areas were determined using the Brunauer–Emmett–Teller (BET) method with a Quantachrome NOVA 3000 Analyzer (USA). The UV–Vis diffuse reflection measurements used a UV–V is (NIR) spectrophotometer (SolidSpec-3700, Shimadzu, Japan). Photoluminescence (PL) measurements were carried out at room temperature using a fluorescence spectrometer (PerkinElmer, LS55, USA) with a xenon lamp as the excitation source (*λ*_ex_ = 370 nm).

### Photocatalytic Activity Measurement

The photocatalytic activity of the samples was investigated by measuring the degradation of methylene blue (MB) in an aqueous solution under simulated sunlight. The light source was a 300-W xenon lamp (PLS-SXE300; 320–780 nm), and the operating current was kept at 15 A. The photon intensity used during photocatalytic activity measurements was 2000 mW cm^−2^ and 10 cm from the outlet of the light source. In this work, 50 mg of pure ZFO nanocrystals, TONTAs (annealed at 600 °C), and ZFO/FTO/TONTAs (annealed at 750 °C) were separately added to 100 mL of an MB aqueous solution (10 mg L^−1^). These suspensions were magnetically stirred for 30 min in the dark to reach absorption–desorption equilibrium, and the MB solution was then replenished to maintain its initial concentration at 10 mg mL^−1^. Subsequently, the suspensions were irradiated under simulated sunlight, and 4-mL aliquots of the suspensions were collected at predetermined intervals (10, 20, 30, 40, 60, 90, 120, and 150 min). The suspensions were centrifuged at 12,000 rpm for 5 min to remove the solid residues, and the concentration of MB molecules in the supernatant was determined by absorbance at 665 nm in the UV–VIS absorption spectrum (UV-1800, Shimadzu, Japan). During photocatalytic MB degradation, the temperature of the sample was kept constant at 25 °C by circulating water.

### Photoelectrochemical Measurement

Photocurrent measurements were carried out in a quartz beaker using an electrochemical workstation (PGSTAT302, Metrohm, Switzerland) in a standard three-electrode configuration with TONTAs and ZFO/FTO/TONTAs as the working electrodes. The counter and reference electrodes were a Pt wire and saturated calomel electrode, respectively. A 0.1 M Na_2_SiO_4_ aqueous solution was used as the electrolyte. The areas of the working electrodes were 12 cm^2^. The working electrode was irradiated with a xenon lamp during the measurements. The distance between the window of the flask and light source was 20 cm. The focused incident light intensity on the flask was ~100 mW cm^−2^.

## Results and Discussion

**Fig. 2:**
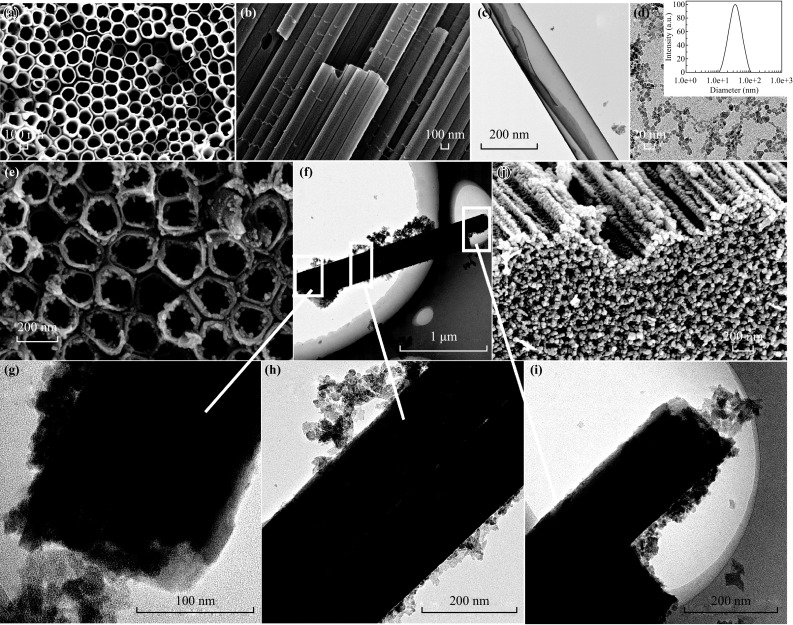
**a** SEM images of the surface morphology and **b** the cross-sectional morphology of the pristine TONTAs. TEM images of the pristine TONTAs **c** and the ZFO nanocrystals **d** (The inset is DLS spectrum of the ZFO nanocrystals). **e** SEM image and **f** TEM image of the pristine ZFO/TONTAs. **g** The left extremity, **h** central part, and **i** right extremity of Fig. **f**. **j** SEM image of the ZFO/FTO/TONTAs annealed at 750 °C

Figure 2a shows that the pristine TONTAs are highly ordered and compactly arranged. The nanotubes are well attached to each other, and the average tube diameter is about 180 nm. The cross-sectional image further illustrates that the pristine TONTAs have a highly oriented structure (Fig. 2b). The nanotubes are straight and cylindrical with an estimated length of 1.8–2.0 μm. The TEM image shows that the diameters of the as-prepared ZFO nanocrystals range from 7 to 15 nm (Fig. 2c), whereas the DLS experiment indicates that *D*_H_ is approximately 35 nm (inset of Fig. 2c). This suggests that weak particle aggregation may exist. Nevertheless, the *D*_H_ of the as-prepared ZFO nanocrystals remains far smaller than the tube diameter of the TONTAs. In fact, Fig. 2d demonstrates that the ZFO nanocrystals could be well perfused into the nanotubes with only a few clogging incidents at the entrance of the nanotubes. Moreover, the gaps between the TiO_2_ nanotubes are filled by ZFO nanocrystals.

The TEM images of the TONTAs before (Fig. 2a, e) and after (Fig. 2c, i) the perfusion of ZFO nanocrystals show that the inner walls of the TONTAs become covered with ZFO nanocrystals, including both extremities and the central area of the nanotubes. This explains why some of the ZFO nanoparticles are observed outside of the nanotubes. After annealing at 750 °C for 2 h, the cross-sectional SEM image shows that the previously smooth walls of the tubes become uneven, which originates from the crystallization of TiO_2_ during annealing (Fig. 2j). The annealed tubes reserve their initial linear arrangement.

**Fig. 3:**
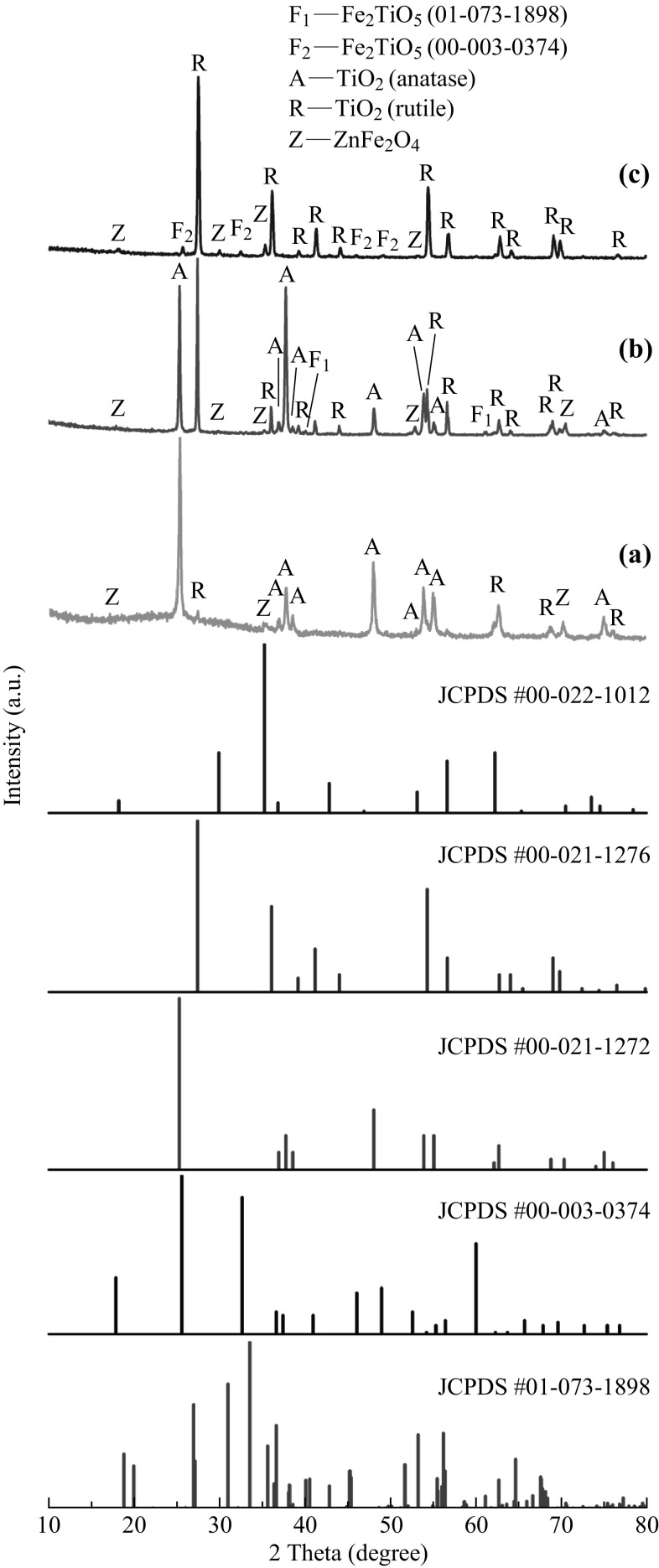
XRD patterns of the ZFO/FTO/TONTAs annealed at **a** 650 °C, **b** 750 °C, and **c** 850 °C

For the as-prepared composite nanotube arrays annealed at 650 °C, anatase TiO_2_ (JCPDS card No. 00-021-1272), rutile TiO_2_ (JCPDS card No. 00-021-1276), and ZFO (JCPDS card No. 00-022-1012) are identified. At 750 °C, in addition to anatase TiO_2_, rutile TiO_2_, and ZFO, diffraction peaks of FTO (JCPDS card No. 01-073-1898) are also observed (Fig. 3a). This demonstrates that the ZFO/FTO/TONTAs were successfully prepared. At 850 °C, anatase TiO_2_ transforms into rutile TiO_2_ (Fig. 3b), while more FTO (JCPDS card No. 00-003-0374) forms at the ZFO/TiO_2_ interface. This suggests that the increase in annealing temperature could promote solid solution reactions. While the anatase phase of TiO_2_ has a higher Fermi level, it also has a lower capacity to absorb oxygen and higher degree of hydroxylation. Thus, it possesses a better photocatalytic activity than rutile TiO_2_ [53]. Nevertheless, a mixture of anatase and rutile is more active than pure anatase [54], including Degussa P25, which is a commercial TiO_2_ photocatalyst. The ZFO/FTO/TONTAs annealed at 750 °C were used for subsequent studies.

**Fig. 4:**
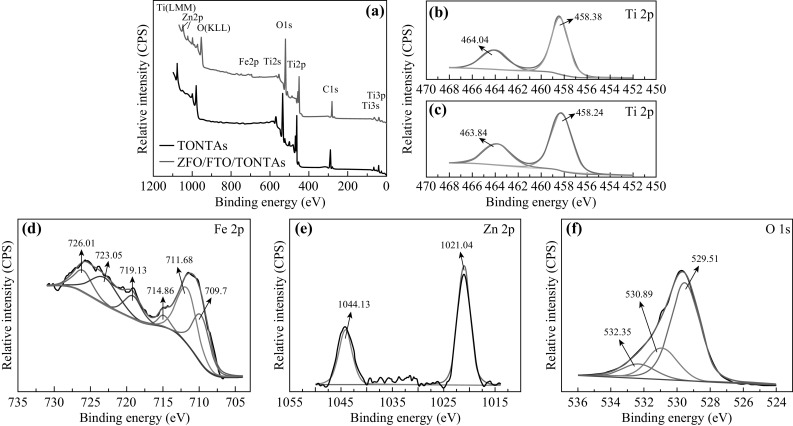
**a** XPS survey spectra of the TONTAs annealed at 600 °C and ZFO/FTO/TONTAs annealed at 750 °C. Ti 2*p* core level XPS spectra of: **b** the TONTAs annealed at 600 °C and **c** ZFO/FTO/TONTAs annealed at 750 °C. XPS spectra of the ZFO/FTO/TONTAs annealed at 750 °C: **d** Fe 2*p*, **e** Zn 2*p*, and **f** O 1*s* core level

Figure 4a shows the XPS survey spectrum of the TONTAs and clearly indicates the presence of all constituent elements, including Ti and O; the C 1*s* peak is ascribed to carbon from the environment. In addition to the peaks corresponding to Ti 2*p* and O 1*s*, Zn 2*p* and Fe 2*p* peaks are found in the XPS survey spectrum of the ZFO/FTO/TONTAs. By observing the 2*p* core level XPS spectra of Ti, the peaks situated at 464.04 and 458.38 eV should be accordingly assigned to Ti 2*p*_1/2_ and Ti 2*p*_3/2_, implying the presence of tetravalent Ti (Ti^4+^) in the TONTAs (Fig. 4b) [48, 55, 56]. However, for the ZFO/FTO/TONTAs, the binding energies of Ti 2*p*_1/2_ and Ti 2*p*_3/2_ are 463.84 and 458.24 eV (Fig. 4c), respectively, suggesting that the Ti 2*p* peaks slightly shift toward lower binding energies, unlike those of the TONTAs. This is attributed to the presence of FTO in the ZFO/FTO/TONTAs. Furthermore, there is no evidence of trivalent Ti (Ti^3+^), which usually appears at 457.4 eV [57].

As presented in Fig. 4d, the Fe 2*p* core level XPS spectrum of the ZFO/FTO/TONTAs can be fitted with six peaks. These peaks are situated at 709.7 and 723.05 eV and represent the +2 oxidation state of iron (Fe^2+^). The peaks at 711.68 and 726.01 eV are assigned to trivalent iron (Fe^3+^). The peaks at 714.86 and 719.13 eV correspond to Fe^2+^ and Fe^3+^ satellite signals, respectively [21, 58, 59]. No Fe^2+^ is observed in the raw materials.

Frandsen et al. [60] reported that Fe^3+^ could be reduced to Fe^2+^ when Ti^4+^ was substituted for Fe^3+^ (2Fe^3+^ → Fe^2+^ + Ti^4+^). Therefore, the solid solution reactions that occur at the interface of ZFO and the pristine TONTAs form Fe^2+^. The Zn 2*p* core level XPS spectrum is shown in Fig. 4e. The peaks situated at 1044.13 and 1021.04 eV are assigned to Zn 2*p*_1/2_ and Zn 2*p*_3/2_, implying the presence of bivalent Zn in the ZFO/FTO/TONTAs [61]. Furthermore, the O 1*s* core level XPS spectrum of the ZFO/FTO/TONTAs (Fig. 4f) can be fitted with three peaks. These peaks are located at 532.35 eV and are ascribed to the presence of the hydroxyl group from absorbed moisture or oxygen vacancy-related defects. The peaks situated at 529.51 and 530.89 eV correspond to lattice oxygen in TiO_2_ and ZFO, respectively [21, 59].

**Fig. 5:**
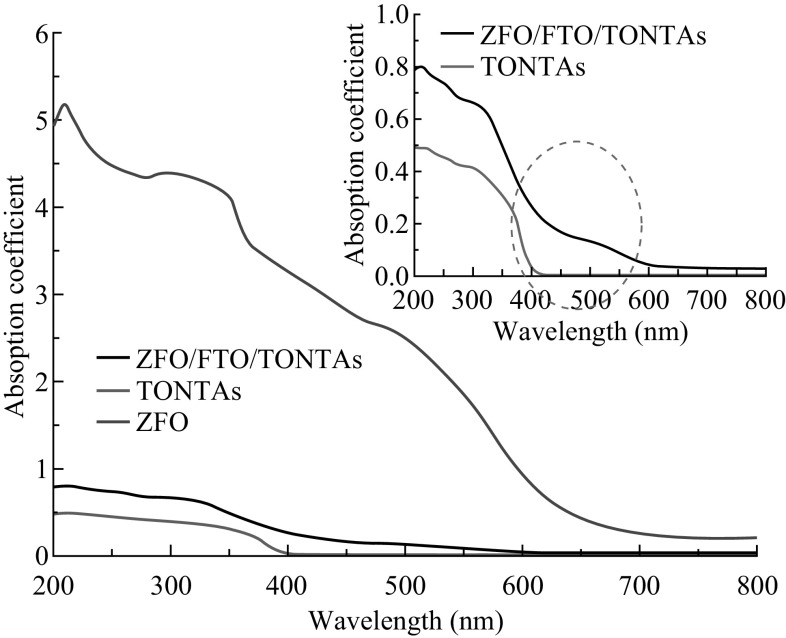
UV–Vis diffuse absorption spectra of the ZFO nanocrystals: TONTAs annealed at 600 °C and ZFO/FTO/TONTAs annealed at 750 °C. The inset shows the corresponding magnified spectra

UV–Vis diffuse reflection was used to investigate the optical properties of the ZFO/FTO/TONTAs, and the optical absorbance was calculated from the optical reflectance data using the Kubelka–Munk function (*α* = (1 − *R*)^2^/2*R*). Here, *α* and *R* are the absorption coefficient and diffuse reflectance coefficient, respectively [62]. As presented in Fig. 5, the ZFO/FTO/TONTAs have a relatively larger *α* than the annealed TONTAs throughout the UV–Vis wavelength range. Its *α* is significantly smaller than that of the pure ZFO nanoparticles. The inset of Fig. 5 indicates that visible light (400–760 nm) absorption of the ZFO/FTO/TONTAs is higher than that of the annealed TONTAs. This might be due to the presence of ZFO and FTO in the heterostructured ZFO/FTO/TONTAs.

**Fig. 6:**
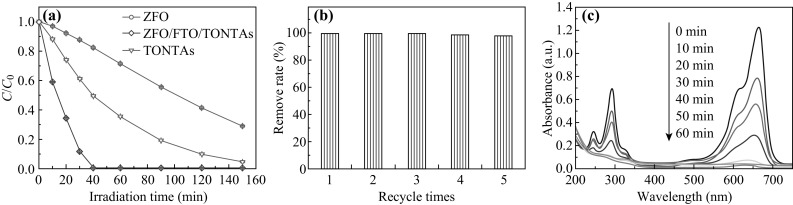
**a** Photocatalytic degradation of MB vs. the irradiation time under simulated sunlight in the presence of the ZFO nanocrystals, TONTAs annealed at 600 °C, and ZFO/FTO/TONTAs annealed at 750 °C. **b** Recycling test for the ZFO/FTO/TONTAs. **c** UV–Vis absorbance of MB as a function of the irradiation time

MB photocatalysis degradation experiments were used to probe the reaction and study the photocatalytic activities of the ZFO/FTO/TONTAs. Figure 6a shows that under simulated sunlight irradiation for 10 min, the ZFO/FTO/TONTAs degrade approximately 41% of MB. About 12% of MB is degraded by the TONTAs, and only 3% of MB degrades in the presence of ZFO under the same conditions. At 40 min of irradiation, the ZFO/FTO/TONTAs could completely degrade the MB molecules. Under the same conditions, the photocatalytic degradation of MB molecules assigned to the TONTAs and ZFO is 51 and 18%, respectively.

The specific surface areas of the ZFO/FTO/TONTAs, TONTAs, and ZFO are 44, 53, and 127.2 m^2^ g^−1^, respectively. In fact, the photocatalytic degradation of MB is achieved through redox reactions that occur at the interface of the photocatalyst and MB molecules. Thus, the specific surface area is an important parameter of the photocatalyst. Nevertheless, while ZFO has a relatively large *α* and specific surface area, its photocatalytic degradation of MB is still very slow. To better compare the photocatalytic efficiency of the above samples, a kinetic study of MB degradation was performed using a pseudo-first-order kinetics model:

1 $$\ln \left( {\frac{{C_{0} }}{C}} \right) = kt$$

where *k* is the apparent reaction constant (min^−1^), and *C*_0_ and *C* are the initial concentration and reaction concentration of MB, respectively. The photocatalytic degradation of MB vs. the irradiation time under simulated sunlight was examined in the presence of the ZFO nanocrystals, TONTAs annealed at 600 °C, and ZFO/FTO/TONTAs annealed at 750 °C (Fig. 6a), and the *k* values are 0.0074, 0.0194, and 0.0646 min^−1^, respectively. These values demonstrate that the ZFO/FTO/TONTAs have a higher efficiency for photocatalytic degradation of MB. Lou et al. reported that the optimal *k* of FTO/TiO_2_ hollow nanospheres was approximately 0.1 min^−1^ when they were used for photocatalytic degradation of rhodamine B [63]. In addition, Xu et al. found that the optimal *k* of a TiO_2_/ZFO photocatalyst was 0.0018 min^−1^ when they were used for photocatalytic degradation of methyl orange [46]. These degradation rates were obtained in different irradiation environments.

As shown in Fig. 6b, the MB removal rate using the ZFO/FTO/TONTAs exhibits a minor decrease (within 3%) after five cycles, which indicates that the ZFO/FTO/TONTAs could remain active and reliable for long-term use. In Fig. 6c, the UV–Vis absorbance demonstrates that the concentration of MB decreases sharply as a function of the irradiation time, which confirms the degradation of MB.

**Fig. 7:**
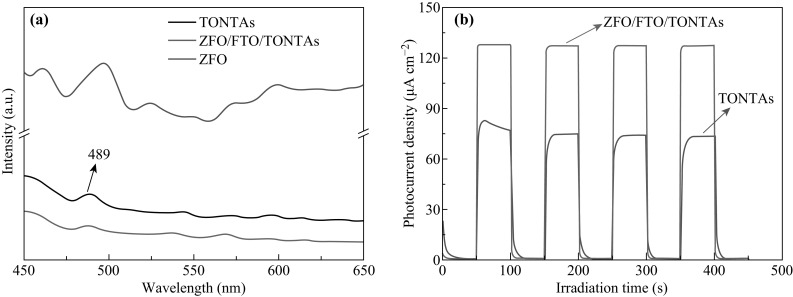
**a** PL spectra of the ZFO, TONTAs, and ZFO/FTO/TONTAs. **b** Photocurrent responses of the TONTAs and ZFO/FTO/TONTAs under simulated sunlight irradiation in a 0.1 M Na_2_SO_4_ solution recorded at 1.0 V. The illumination was interrupted every 50 s

PL spectra are commonly used to investigate the separation efficiency of photogenerated electron–hole pairs in a semiconductor because recombination of electron–hole pairs produces a PL emission signal [64]. Figure 7a shows that the peaks in the PL spectra (near 489 nm) sharply decrease for the ZFO/FTO/TONTAs with respect to those of ZFO and the TONTAs, which indicates efficient separation of the photogenerated electron–hole pairs. Furthermore, it explains why the ZFO/FTO/TONTAs show higher photocatalytic degradation efficiency. To further prove the effective charge separation of the ZFO/FTO/TONTAs, electrochemical analysis was carried out. The current–time (*I*-*t*) characteristics of the TONTA and ZFO/FTO/TONTA electrodes recorded in 0.1 M Na_2_SO_4_ under simulated sunlight irradiation are shown in Fig. 7b. The photocurrent density of the ZFO/FTO/TONTAs is much higher than that of the TONTAs, which further confirms that ZFO/FTO/TONTAs have a higher separation efficiency of photogenerated electron–hole pairs.

**Fig. 8:**
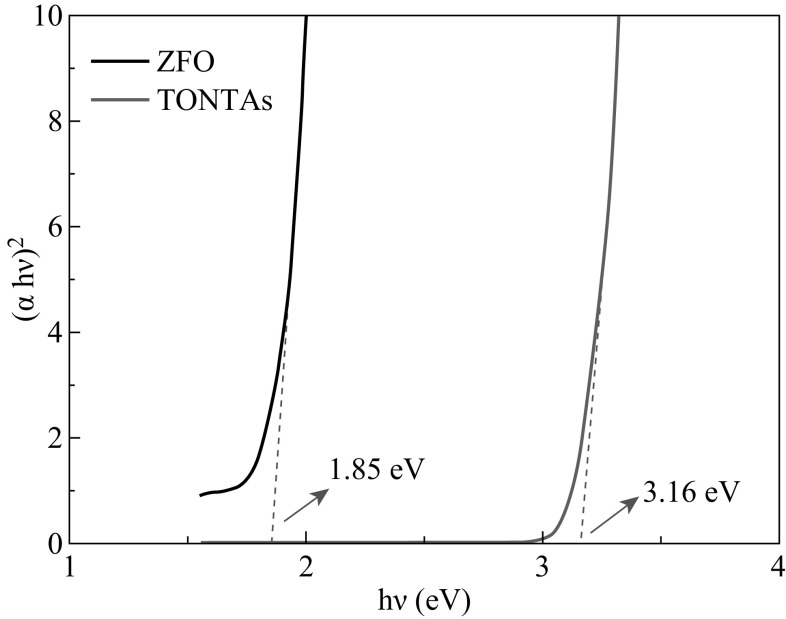
Plots of (*αhv*)^2^ versus the incident photon energy that are assigned to the as-prepared ZFO and TONTAs

According to the Kubelka–Munk function and the plot of (*αhν*)^2^ against the energy of absorbed light (*hν*), the bandgaps (*E*_g_) of ZFO and the TONTAs are estimated as 1.85 and 3.16 eV, respectively (Fig. 8). Courtin et al. [50] reported that the *E*_g_ of FTO was approximately 2.2 eV. *E*_CB_ and *E*_VB_ represent the band edge potentials of the conduction band (CB) and valence band (VB), respectively. These can be calculated from the following equations [65]:

2 $$E_{\text{CB}} = X{-}E^{C} {-}0.5E_{\text{g}}$$

3 $$E_{\text{VB}} = X{-}E^{C} + \, 0.5E_{\text{g}}$$

where *X* is the electronegativity of the semiconductor, which is the geometric mean of the electronegativity of the constituent atoms, and *E* ^*C*^ is the energy of the free electrons on the hydrogen scale (approximately 4.5 eV). Moreover, the *X* values for ZFO, FTO, and TiO_2_ are 5.05, 5.86, and 5.81 eV, respectively [50, 61, 66]. Based on Eqs. 1 and 2, the *E*_CB_ values of ZFO, FTO, and TiO_2_ are separately estimated to be − 0.375, 0.26, and − 0.27 eV/normal hydrogen electrode (NHE). Their corresponding *E*_VB_ values are 1.475, 2.46, and 2.89 eV/NHE.

**Fig. 9:**
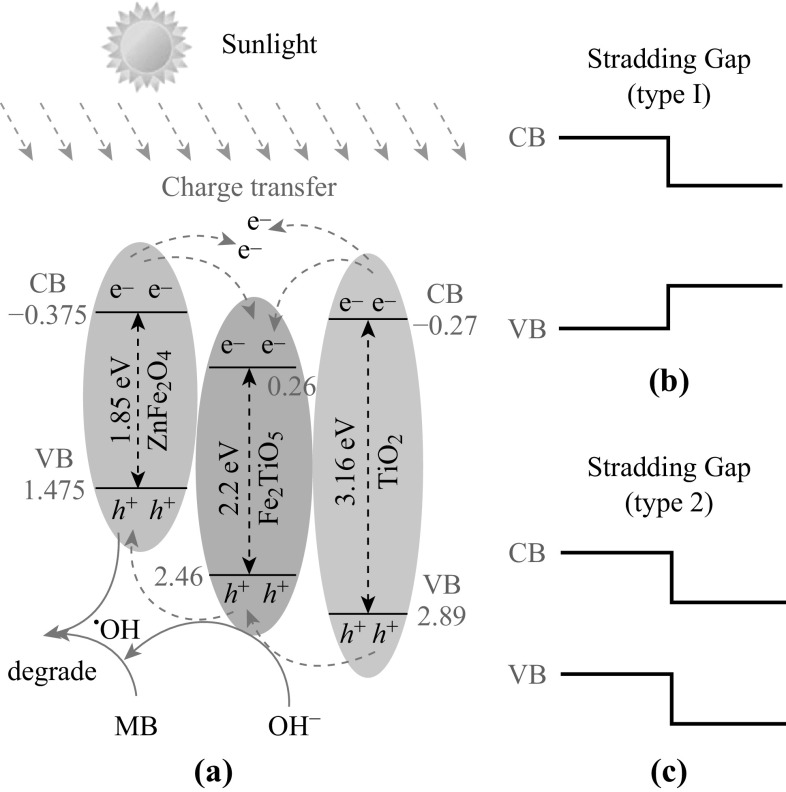
Schematic of the energy band structure of **a** the ZFO/FTO/TONTAs heterojunction, **b** type I band alignment, and **c** type II and alignment

The ZFO/FTO/TONTAs consist of three different semiconductors (ZFO, FTO, and TiO_2_), and two different heterojunctions are formed in the ZFO/FTO/TONTAs. As depicted in Fig. 9a, the CB of FTO lies below that of TiO_2_ and ZFO, and the VB of ZFO lies above that of FTO and TiO_2_. This produces a staggered type II band alignment between ZFO and FTO, while a type I band alignment is produced between FTO and TiO_2_. This implies the coexistence of type I and staggered type II band alignments in ZFO/FTO/TONTAs. The photogenerated electrons present in the CB of ZFO at the ZFO/FTO interface—with a staggered type II band alignment—are transferred to the CB of FTO, while the holes present in the VB of FTO are transferred to the VB of ZFO. This facilitates separation of photogenerated electrons and holes. However, because of formation of type I band alignment at the FTO/TiO_2_ interface, the photogenerated electrons present in the CB of TiO_2_ are transferred to the CB of FTO, and the holes present in the VB of TiO_2_ are also transferred to the VB of FTO. In this case, the photogenerated electrons and holes easily recombine. Nevertheless, the holes that are originally transferred from the VB of TiO_2_ to the VB of FTO can continue to be transferred to the VB of ZFO because there is a ZFO/FTO heterojunction in the ZFO/FTO/TONTAs. Thus, they are reduced, and the photogenerated electrons recombine with holes at the FTO/TiO_2_ interface.

Lotgering et al. [67] demonstrated the existence of electron exchange between Fe^2+^ and Fe^3+^ via paramagnetic Mossbauer spectroscopy to test Ti-doped ZFO. Figure 4d shows that Fe^2+^ and Fe^3+^ coexist in the ZFO/FTO/TONTAs, and this implies that the photogenerated electrons can be transferred at the ZFO/FTO interface by Fe^2+^/Fe^3+^ electron exchange. In addition, the unique axially oriented structure of the ZFO/FTO/TONTAs also facilitates electron transfer, supporting the view that the photogenerated electrons and holes can be effectively separated. Furthermore, the holes on the surface of the ZFO could reduce H_2_O or OH^−^ to $$^{{ \cdot }} {\text{OH}}$$ because its energy (1.475 eV vs. NHE) is higher than the standard redox potential of *E* $$\left( {{\raise0.7ex\hbox{${{\text{OH}}^{\text{ - }} }$} \!\mathord{\left/ {\vphantom {{{\text{OH}}^{\text{ - }} } {^{{ \cdot }} {\text{OH}}}}}\right.\kern-0pt} \!\lower0.7ex\hbox{${^{{ \cdot }} {\text{OH}}}$}}} \right) = 1.99\,\,\,{\text{eV}}$$ (vs. NHE). Strongly oxidative $$^{{ \cdot }} {\text{OH}}$$ could degrade MB. Thus, the improved photocatalytic degradation efficiency seen in the ZFO/FTO/TONTAs is mainly attributed to the following points: (1) enhanced visible light absorption from the introduction of ZFO and (2) more effective separation of photogenerated electrons and holes because of the coexistence of type I and staggered type II band alignments in the ZFO/FTO/TONTAs.

## Conclusions

In this work, ZFO nanocrystals were successfully perfused into the TONTA pipelines using a bias voltage-assisted perfusion method. After annealing at 750 °C for 2 h, heterostructured ZFO/FTO/TONTAs were obtained. This formed a staggered type II band alignment at the ZFO/FTO interface and a type I band alignment at the FTO/TiO_2_ interface. Because of the singular nanoscale heterostructure, the visible light absorption of the ZFO/FTO/TONTAs was greatly enhanced upon introduction of ZFO and FTO. Despite the small specific surface area, the efficiency of the ZFO/FTO/TONTAs in the photocatalytic degradation of MB was significantly improved upon irradiation with simulated sunlight with a reliable recycling ability.
